# Optimizing Venous Thromboembolism Prophylaxis in Achilles Tendon Rupture: A Systematic Review of Current Evidence and Protocol Recommendations

**DOI:** 10.7759/cureus.95184

**Published:** 2025-10-22

**Authors:** Mohamed Hashem, Mohamed Elbeshbeshy, Mohamed Khalafallah, Yousif A Othman, Ahmed Al-Saqili, Ahmed A Ghobashy

**Affiliations:** 1 Orthopaedics, Frimley Health NHS Foundation Trust, London, GBR; 2 Trauma and Orthopaedics, Wirral University Teaching Hospital, Wirral, GBR; 3 Medicine, Faculty of Medicine, Alexandria University, Alexandria, EGY; 4 Trauma and Orthopaedics, Faculty of Medicine, Fayoum University, Fayoum, EGY; 5 Trauma and Orthopaedics, Faculty of Medicine, Alexandria University, Alexandria, EGY

**Keywords:** achilles tendon injury, achilles tendon ruptures, deep vein thrombosis (dvt), venous thromboembolism (vte), vte prophylaxis

## Abstract

The Achilles tendon plays a vital role in maintaining normal gait and locomotion. The incidence of Achilles tendon rupture (ATR) has been increasing, mainly due to the rising popularity of recreational sports. This review assesses anticoagulation efficacy in preventing venous thromboembolism (VTEs) during ATR treatment and aims to establish an evidence-based prophylaxis protocol. Initially, a comprehensive search across PubMed, Scopus, Web of Science (WoS), and the Cochrane Library databases, and the citation analysis identified studies on VTE prophylaxis in patients with acute or chronic ATR. We summarized results qualitatively, focusing on VTE prevention, complications, and clinical outcomes while considering study quality, bias, and confounders in our analysis. Eight studies, involving 1199 patients with ATR, were analyzed for the efficacy of various VTE prophylaxis strategies. Risk stratification using validated tools, such as the Thrombosis Risk Prediction Following Cast Immobilization (TRiP(cast)) score, was consistently advocated. High-risk patients benefited substantially from pharmacological prophylaxis, typically low molecular weight heparin (LMWH) or rivaroxaban, for a minimum duration of 28 days. In contrast, patients assessed as low-risk effectively managed VTE risks through early mobilization, with supplementary mechanical prophylaxis when indicated. The importance of individualized monitoring for VTE symptoms, bleeding complications, and patient adherence was also highlighted, particularly addressing obesity, prior VTE episodes, pregnancy, and the type of surgical intervention. The findings from this review advocate for an individualized approach to VTE prophylaxis in Achilles tendon rupture patients. Employing structured risk assessments using validated tools, e.g., TRiP(cast) score, ensures targeted and effective prophylactic interventions, significantly reducing complications. Pharmacological prophylaxis (LMWH or rivaroxaban) is indicated in high-risk patients; however, its benefit in low-risk patients remains uncertain and inconsistently demonstrated across studies. In these patients, early mobilization, patient education, and mechanical prophylaxis may offer a safe alternative. Further high-quality, long-term studies are needed to optimize prophylaxis duration and refine patient-specific protocols, especially in low-risk patients, ultimately improving patient outcomes and safety in clinical practice.

## Introduction and background

The Achilles tendon is the largest and most robust tendon in the human body. It connects the calf muscles to the calcaneus bone and plays a crucial role in locomotion, including walking, running, jumping, and ascending stairs [1].

The incidence of Achilles tendon injury has been increasing, mainly due to the rising popularity of recreational sports, which has led to a higher prevalence of Achilles tendon rupture (ATR). The overall incidence of ATR in the general population is approximately 31 cases per 100,000 individuals annually, though this rate may be higher in specific populations and regions [2]. The male-to-female incidence ratio is approximately 5.5:1, with over 80% of ruptures occurring during recreational sports [3].

Management options for ATR include surgical intervention followed by immobilization with a brace or plaster cast, as well as conservative treatment involving immobilization alone [4-6]. A significant complication associated with ATR treatment is venous thromboembolism (VTE), a well-documented cause of morbidity and mortality in lower limb surgery and prolonged immobilization [4,7,8]. VTEs account for about 10% of all hospital deaths [8,9]. Reported rates of deep vein thrombosis (DVT) following ATR treatment range from 0.43% to 34%, while pulmonary embolism (PE) rates vary from 0% to 3% [10,11].

Previous studies have suggested the use of low molecular weight heparin (LMWH) as prophylaxis to reduce the incidence of VTE events following temporary lower limb immobilization [6,12]. However, findings across studies have been inconsistent, and there is currently no consensus on the necessity or efficacy of prophylactic therapy following ATR, especially in the lower risk group [4,7,13,14].

Therefore, this systematic review aims to evaluate the efficacy and safety of anticoagulation therapy in preventing VTEs in patients undergoing ATR treatment and to establish an evidence-based protocol for using prophylactic anticoagulants in patients with ATR.

## Review

Methods

This systematic review was conducted by the Preferred Reporting Items for Systematic Reviews and Meta-Analyses (PRISMA 2020) guidelines [15]. The study protocol was prospectively registered in the International Prospective Register of Systematic Reviews (PROSPERO; registration number: CRD420251108036).

Literature Search and Study Selection

A comprehensive literature search was performed across multiple databases, including PubMed, Scopus, Web of Science (WoS), and the Cochrane Library, to identify relevant studies published within the past 20 years. A tailored search strategy was developed for each database, as detailed in Appendix A. Duplicate records were removed using EndNote (Clarivate, Philadelphia, PA) [16], and both prospective and retrospective citation analyses were conducted through manual searches.

Inclusion and Exclusion Criteria

Studies were included if they met the following criteria: (a) involved patients diagnosed with acute or chronic ATR; (b) reported on VTE prophylaxis strategies and associated clinical outcomes; (c) consisted of clinical trials or well-designed observational studies; and (d) were published in English with full-text availability. Studies were excluded if they met any of the following criteria: (a) published in languages other than English; (b) assessed as low quality based on study design or methodological limitations; or (c) consisted of cross-sectional studies, case series, case reports, review articles, cadaveric studies, or conference abstracts.

Study Selection Process

We conducted the study selection in two stages. First, we screened titles and abstracts, and then we evaluated full texts for eligibility. Two reviewers performed each stage independently and resolved any disagreements through discussion with the supervising author.

Data Extraction

Data extraction was performed independently by two authors using standardized spreadsheets. The following variables were collected from each included study: study design, country of study, population characteristics, VTE prophylaxis regimen, total sample size, follow-up duration, key findings, mean age, proportion of male participants, body mass index (BMI), smoking status, prevalence of diabetes mellitus, history of VTE, and site of rupture.

Quality Assessment

The risk of bias for each included study was assessed by two independent reviewers using appropriate tools based on the study design. Randomized controlled trials (RCTs) were evaluated using the Cochrane Risk of Bias 2 (RoB 2) tool [17]. Cohort studies were assessed using the Newcastle-Ottawa Scale (NOS) [18]. Any disagreements in quality assessment were resolved through discussion between the reviewers or by the senior author.

Narrative Synthesis

A narrative synthesis was conducted to integrate and interpret the findings from the included studies. The synthesis followed a structured approach, evaluating study characteristics, methodological quality, and primary outcome measures. Findings were grouped based on study design, patient demographics, and the type of VTE prophylaxis implemented. Patterns, similarities, and differences across studies were identified to provide a comprehensive overview of the current evidence. Where applicable, results were qualitatively summarized, with emphasis on trends in VTE prevention strategies, complication rates, and clinical outcomes. The synthesis considered study quality, potential biases, and confounding factors. Notable differences among studies (such as variations in follow-up duration, intervention methods, and patient populations) were also highlighted.

Results

**Figure 1:**
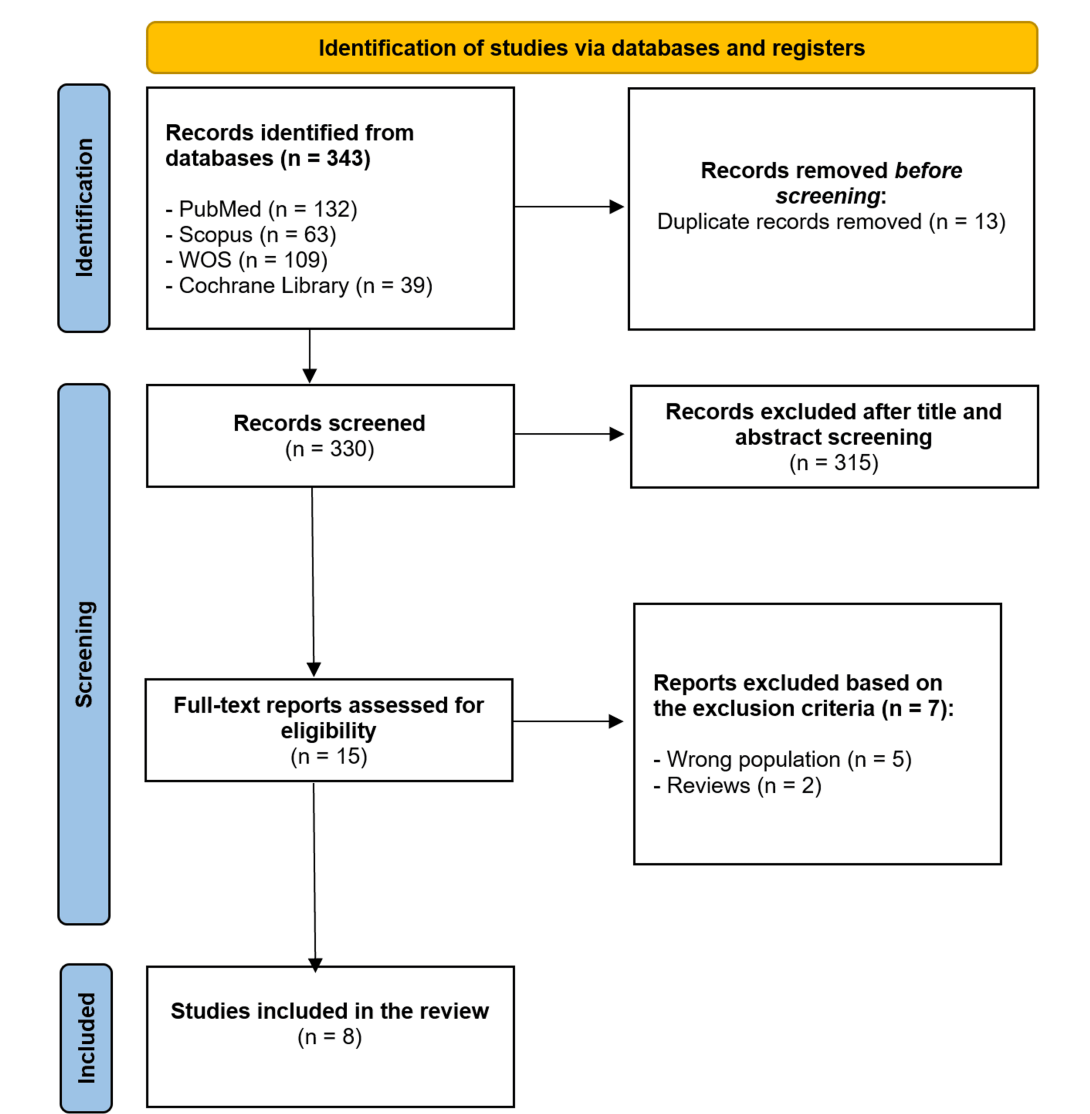
A PRISMA flowchart of the study selection process PRISMA: Preferred Reporting Items for Systematic Reviews and Meta-Analyses; WoS: Web of Science

The initial search retrieved 343 studies. After removing 13 duplicates, we screened the titles and abstracts of 330 studies. Then, we excluded 315 articles, and only 15 studies were screened for eligibility. Finally, we included eight studies consisting of four clinical trials and four cohort studies (Figure 1).

Characteristics of the Included Studies

The included studies encompassed a total of 4,701 patients, of whom 1,199 were diagnosed with ATR. The studies were conducted across various countries, including the United Kingdom, Turkey, and South Africa. Follow-up durations ranged from 35 days to 12 months. A range of prophylactic anticoagulation regimens was employed, including LMWH formulations such as dalteparin, tinzaparin, nadroparin, and reviparin, as well as rivaroxaban and macrodex (Tables 1-4).

**Table 1:** Summary of the included studies UK: United Kingdom; ATR: Achilles tendon rupture; RCT: randomized controlled trials; LMWH: low molecular weight heparin; NWB: non-weight-bearing; EWB: early weight-bearing; FWB: full weight-bearing; PE: pulmonary embolism; CAF: conservatively-managed ankle fractures; SAF: surgically-managed ankle fractures; US: ultrasound

Author, year published	Design	Country	ATR Population	Prophylactic anticoagulation, Duration	No of ATR	Follow-up Duration	VTE risk (incidence/group size)	VTE screening	Findings
Blanco et al., 2017 [4]	Prospective cohort study	UK	ATR managed with a functional walker boot protocol and FWB for eight weeks.	LMWH (dalteparin) until the end of immobilization	283	Six weeks	4.9% (14/283)	Symptomatic only	Low overall incidence of VTE in foot and ankle trauma (3.5%). No statistically significant difference in VTE incidence among the ATR group compared to surgically or conservatively managed ankle fracture groups (4.9% vs. 3% vs. 2.2% respectively; p=.197). However, VTE occurred significantly earlier following acute ATR vs. ankle fractures (16.1 vs. 35.5 days, p=.002). VTE incidence in high-risk patients despite receiving LMWH varied between 33% (one in three high-risk patients) in the ATR group, 14.3% (1/7) in the CAF group, and 0% (0/4) in the SAF group.
Çolak et al., 2020 [6]	Retrospective cohort study	Turkey	Surgically managed ATR	LMWH (enoxaparin, 4000 U) for 20 days postoperatively	238	12 weeks	9.2% (22/238)	Symptomatic only	18 DVTs and four PEs. Risk factors were age >40 years and BMI >30 kg/m2. Hence, the recommendation for routine VTE prophylaxis in acute ATR.
Lapidus et al., 2007 [13]	RCT	Sweden	Surgically repaired ATR – postoperative: a) Placebo	Six weeks postoperatively (both groups)	91	Six weeks	36% (16/44)	Duplex US at three and six weeks for all patients	High incidence of VTE following surgical repair of ATR. VTE prophylaxis using dalteparin was not statistically protective compared to placebo (p=.8).
			b) LMWH (dalteparin 5000 U)				34% (16/47)		
Lee et al., 2023 [7]	Retrospective cohort study	UK	Patients ≥18 years old with acute ATR: a) EWB	LMWH (Tinzaparin) for four weeks (both groups)	296	Nine weeks	1.3% (2/69)	Symptomatic only	Relatively lower VTE incidence in EWB vs. NWB groups, but was not statistically significant (p=.33).
			b) NWB				2.9% (3/227)		
Nemeth et al., 2019 [19]	Validation RCT	Netherlands	Acute ATR: a) Surgical repair	LMWH (nadroparin or dalteparin) till the end of immobilization	94	12 weeks	19% (4/21)	Symptomatic only	The relative risk of VTE following surgical repair versus conservative treatment was 3.45. In an adjusted analysis, the relative risk was 8.2 (95% CI: 2.8–24.1).
			b) Cast immobilization				5.5% (4/73)		
Nilsson-Helander et al., 2009 [14]	RCT	Sweden	Unilateral ATR in patients 16–65 years old if randomisation and treatment were started within 72 hours from the initial injury: a) Surgically-managed	Macrodex	95	Eight weeks	28.6% (14/49)	Duplex US at eight weeks for all patients	High overall VTE incidence of 34% (32/95) with no statistically significant difference between the groups (p=.217). Three of the 32 patients experienced non-fatal PE in addition to DVTs. In the higher risk subgroup (n=7), prophylactic anticoagulation (dalteparin) resulted in a lower VTE incidence of 14% (one VTE event in the seven patients).
			b) Non-surgical in a below-knee cast for two weeks, followed by a functional brace for 6 weeks.	LMWH (based on surgeon’s preference)			39.1% (18/46)		
Saragas et al., 2017 [20]	Prospective cohort study	South Africa	ATR repair followed by NWB in a below-knee cast for ≥ 4 weeks in patients >18 years old	Rivaroxaban 10 mg OD for 4 weeks	28	One year	3.6% (1/28)	Symptomatic only	The only incident occurred at five weeks; however, the patient admitted to being non-compliant with taking his rivaroxaban.
Walenga et al., 2014 [12]	Validation study of a previous RCT	Denmark	ATR requiring functional (ambulatory) immobilization in a plaster cast or brace	LMWH (reviparin) vs placebo for 5-6 weeks	74	35-42 days	12.2% (9/74)	Ascending phlebography at the time of brace removal in all patients	Six events in the placebo group vs. three events in the reviparin group

**Table 2:** Baseline charachterstics of included RCTs (all populations including non-ATR patients) NA: not applicable; LMWH: low molecular weight heparin; ATR: Achilles tendon rupture; RCT: randomized controlled trials

Author, year published	Groups	Participants (N)	Mean age, years (SD)	Male, n (%)	Body mass index, kg/m^2^ (SD)	Smoking, n (%)	Diabetes mellitus, n (%)	History of venous thromboembolism, n (%)	Side rupture, n (%)	
									Right	Left
Lapidus et al., 2007 [13]	LMWH dalteparin	52	37 (8)	41 (79)	26 (3)	9 (17.31)	0	0	NA	NA
	Placebo	53	42 (9)	42 (79)	26 (3)	8 (15.09)	0	0	NA	NA
Nemeth et al., 2019 [19]	LMWH	719	46.5 (16.5)	347 (48.3)	26.0 (4.4)	188 (28.4)	NA	NA	NA	NA
	No treatment	716	45.6 (16.4)	369 (51.5)	25.7 (4.4)	178 (24.9)	NA	NA	NA	NA
Nilsson-Helander et al., 2009 [14]	ATR, Marcodex or LMWH	95	42.25 (7.86)	79 (83.15)	NA	NA	0	NA	NA	NA
Walenga et al., 2014 [12]	LMWH (reviparin)	217	46.33 (13.43)	112 (52)	25.33 (3.73)	79 (36)	5 (2)	5 (2)	NA	NA
	Placebo	221	46.67 (14.18)	114 (52)	26 (2.98)	103 (47)	5 (2)	5 (2)	NA	NA

**Table 3:** Baseline characterstics of included non-randomised studies (all populations including non-ATR patients) NA: Not applicable; LMWH: low molecular weight heparin; ATR: Achilles tendon rupture

Author, year published	Groups	Participant (N)	Mean age, years (SD)	Male, n (%)	Body mass index, kg/m^2^ (SD)	Smoking, n (%)	Diabetes mellitus, n (%)	History of venous thromboembolism, n (%)	Side rupture, n (%)	
									Right	Left
Lapidus et al., 2007 [13]	LMWH dalteparin	52	37 (8)	41 (79)	26 (3)	9 (17.31)	0	0	NA	NA
	Placebo	53	42 (9)	42 (79)	26 (3)	8 (15.09)	0	0	NA	NA
Nemeth et al., 2019 [19]	LMWH	719	46.5 (16.5)	347 (48.3)	26.0 (4.4)	188 (28.4)	NA	NA	NA	NA
	No treatment	716	45.6 (16.4)	369 (51.5)	25.7 (4.4)	178 (24.9)	NA	NA	NA	NA
Nilsson-Helander et al., 2009 [14]	ATR, Marcodex or LMWH	95	42.25 (7.86)	79 (83.15)	NA	NA	0	NA	NA	NA
Walenga et al., 2014 [12]	LMWH (reviparin)	217	46.33 (13.43)	112 (52)	25.33 (3.73)	79 (36)	5 (2)	5 (2)	NA	NA
	Placebo	221	46.67 (14.18)	114 (52)	26 (2.98)	103 (47)	5 (2)	5 (2)	NA	NA

**Table 4:** Baseline characterstics of the study by Saragas et al., 2017 NA: not applicable; ORIF: open reduction internal fixation; NWB: non-weight bearing; OATS: osteochondral autogenous transplantation system

Author, year published	Groups	Participant (N)	Mean age, years (SD)	Male, n (%)	Body mass index, kg/m^2^ (SD)	Use of steroids, n (%)	Smoking, n (%)	Diabetes mellitus, n (%)	History of venous thromboembolism, n (%)	Side rupture, n (%)	
										Right	Left
Saragas et al., 2017 [20]	Hindfoot arthrodesis	22	50.9 (11.542)	75 (52.82)	29 (7.363)	NA	14 (9.9)	NA	0	NA	NA
	Mid-foot arthrodesis	19									
	Acquired flat-foot correction	18									
	Achilles tendon rupture repair	17									
	Ankle arthrodesis + tibiotalocalcaneal arthrodesis	16									
	Achilles tendon debridement and repair	11									
	Tendon (other than Achilles tendon) debridement/repair	7									
	Total ankle replacement	7									
	Lateral ligament reconstruction	5									
	Dwyer calcaneal osteotomy	4									
	Ankle fracture ORIF	4									
	OATS	2									
	Lisfranc ORIF	2									
	Jones fracture ORIF	2									
	Gastrocnemius recession	2									
	Dislocating peroneal tendons	2									
	Tendon transfers	1									
	Non-union tibia (revision)	1									

Risk of Bias Assessment

**Figure 2:**
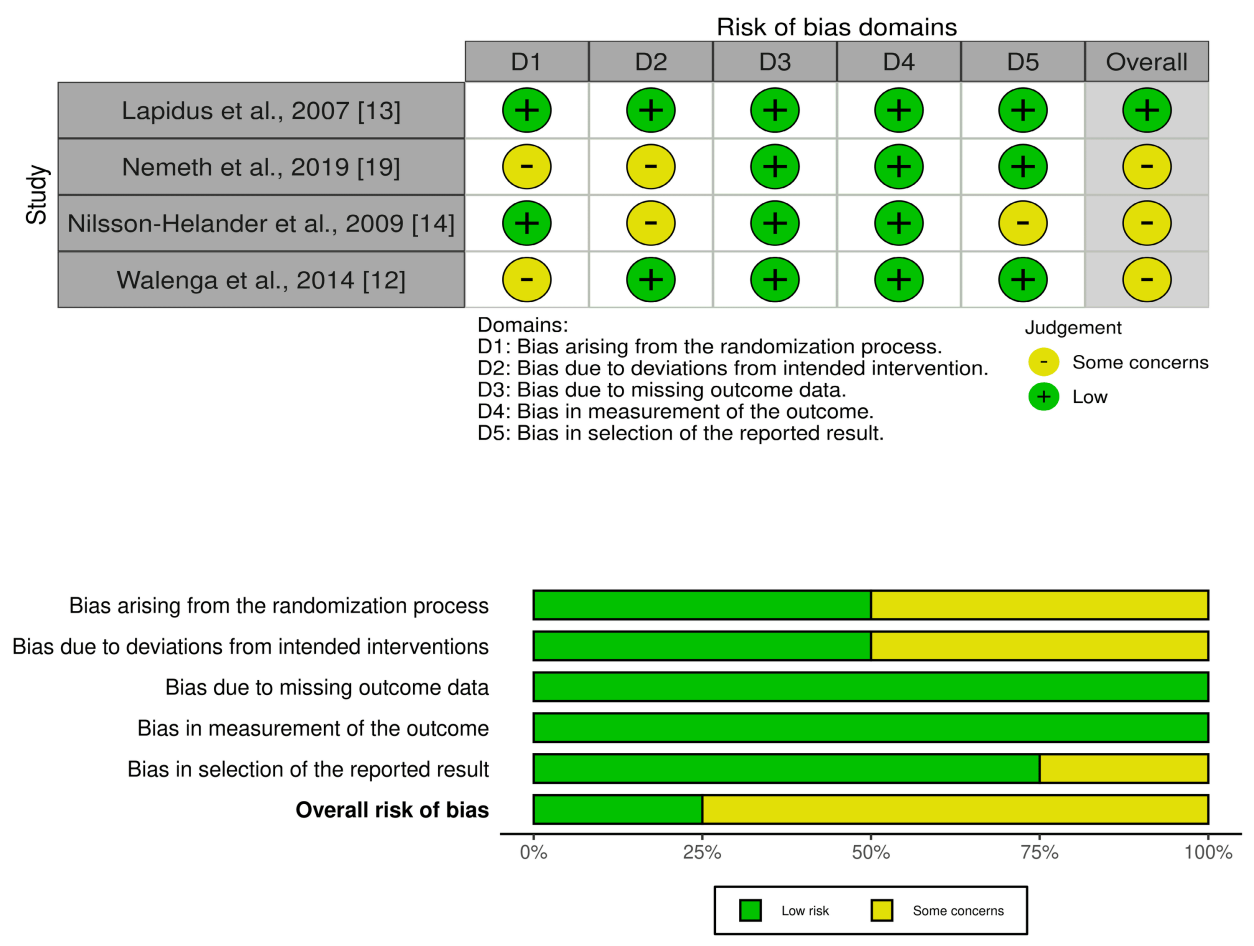
Risk of bias assessment of the included RCTs RCTs: randomized controlled trials

Regarding the RCTs, one study had a low risk of bias [13], and the other three studies were rated as "some concerns" (Figure 2) [12,14,19].

The four included non-randomised studies were classified as illustrated in Table 5.

**Table 5:** Newcastle-Ottawa Scale (NOS) quality assessment of included non-randomised studies

Author, year published	Selection		Comparability	Outcomes	Total scores
Blanco et al., 2017 [4]	4	2		3	9 - Good
Colak et al., 2020 [6]	3	2		3	8 - Good
Lee et al., 2023 [7]	4	2		2	8 - Good
Saragas et al., 2017 [20]	2	0		2	4 - Fair

Discussion

This systematic review synthesized data from eight studies evaluating VTE prophylaxis in patients with ATR, spanning both surgical and non-surgical management. The studies varied in design, pharmacologic agents, duration of treatment, and patient populations, which limits direct comparability but provides a comprehensive view of current prophylactic strategies and their outcomes.

Prophylactic Agent Efficacy

LMWH was the most commonly studied pharmacologic agent. Blanco et al. [4] and Lee et al. [7] reported low VTE incidences of 4.9% and 1.7%, respectively, with LMWH prophylaxis administered for 28 days or during immobilization, with no significant bleeding complications noted. Similarly, Walenga et al. [12] reported an overall VTE risk of 12% (nine VTEs in 74 ATR patients). Among them, three (33.3%) were in the reviparin group vs. six (66.7%) in the placebo cohort, suggesting a possible reduction in events with LMWH.

In contrast, Lapidus et al. [13] and Nilsson-Helander et al. [14] found no significant difference in VTE incidence between dalteparin and placebo groups (approximately 34% vs. 36%), raising questions about the routine use of LMWH for all ATR patients. Likewise, Nemeth et al. [19] reported no statistically significant benefit of LMWH in preventing VTE among cast-immobilized or surgically repaired ATR patients, further questioning its utility in low-risk or non-surgical cases.

Saragas et al. [20] evaluated rivaroxaban in 28 post-surgical ATR patients. They reported only one symptomatic VTE (3.6%) occurring in a patient who admitted non-compliance, suggesting favorable outcomes with direct oral anticoagulants (DOACs) if adherence is maintained.

Duration of Prophylaxis

Prophylaxis duration varied across studies from 20 days to six weeks. Blanco et al. [4], Lee et al. [7], and Walenga et al. [12] administered LMWH or reviparin for 28-42 days, reporting reduced VTE rates. Similarly, Saragas et al. [20] employed rivaroxaban for four to six weeks with a low VTE incidence. However, Nemeth et al. [19] used a 28-day LMWH regimen, and Çolak et al. [6] used a 20-day enoxaparin regimen but still reported an 8.5% and 9.1% VTE rate, respectively, suggesting that duration alone may be insufficient without appropriate risk stratification. Nilsson-Helander et al. [14] left prophylaxis duration to the surgeon’s discretion, which may have contributed to inconsistent outcomes.

Risk Factors and Surgical Impact

Surgical intervention and prolonged immobilization were consistently associated with higher VTE risk. Nemeth et al. [19] identified surgical repair as conferring an 8.2-fold increased VTE risk (relative risk (RR) 8.2). Nilsson-Helander et al. [14] reported a 34% VTE rate in surgically treated patients; however, the difference was not statistically significant compared to the non-surgically managed group. Çolak et al. [6] observed a 9.1% postoperative VTE rate despite LMWH prophylaxis. Conversely, Blanco et al. [4] and Lee et al. [7] emphasized the importance of risk stratification and early weight-bearing protocols, respectively, to reduce VTE incidence.

Safety and Monitoring

Across all studies, bleeding complications were rare. Lapidus et al. [13], Blanco et al. [4], and Nemeth et al. [19] found no significant bleeding or heparin-induced thrombocytopenia. Nilsson-Helander et al. [14] noted non-thrombotic complications related primarily to surgery, such as wound infection. Saragas et al. [20] and Çolak et al. [6] reported no major adverse events, supporting the general safety of anticoagulants when appropriately selected and monitored.

VTE Prophylaxis Protocol for Patients With ATR

Based on current evidence, a structured, individualized approach to VTE prophylaxis is recommended for patients with ATR, incorporating risk stratification, agent selection, duration of therapy, and ongoing monitoring.

Step 1: initial assessment and risk stratification: Clinicians should use a validated prediction model for VTE risk, such as the Thrombosis Risk Prediction Following Cast Immobilization (TRiP(cast)) score, in patients with lower limb immobilization [21,22]. A score ≥7 identifies high-risk patients warranting pharmacologic prophylaxis. ATR automatically confers 3 points due to its classification as high-risk trauma. This is supported by Calder et al. [23] findings that both surgically and conservatively managed ATRs conferred a higher clinical and radiological VTE risk compared to other isolated foot and ankle injuries. Additional risk factors in the TRiP(cast) score include age >35 years, male sex, BMI >25 kg/m^2^, personal or first-degree family history of VTE, recent surgery or immobilization, hormonal therapy, malignancy, pregnancy or puerperium, and comorbidities such as congestive heart failure (CHF), chronic kidney disease (CKD), chronic obstructive pulmonary disease (COPD), rheumatoid arthritis, and varicose veins [21,22].

Step 2: prophylactic agent selection: For high-risk or surgical patients, LMWH (including enoxaparin, dalteparin, and reviparin) remains the most commonly used agent [4,6,7,12]. Rivaroxaban, an oral direct anticoagulant, can be an alternative for patients preferring oral therapy, provided adherence is ensured [20]. In low-risk patients, evidence remains inconclusive due to the high overall VTE incidence rates following ATR, with some studies reporting a substantial reduction in VTE risk with LMWH compared to placebo [6,12]. However, most studies do not support routine pharmacologic prophylaxis. Lapidus et al. [13] and Nilsson-Helander et al. [14] found no significant reduction in VTE risk with LMWH in this group. Instead, they advocated for early mobilization, weight-bearing protocols, and mechanical prophylaxis when mobility is limited, which aligns with the general lower limb immobilization findings by Douillet et al. [22]. Hence, a personalized shared decision-making approach is required in these cases.

Step 3: duration of prophylaxis: A minimum of 28 days of prophylaxis is advised in high-risk or surgically treated patients [4,7,12]. Some evidence supports extending prophylaxis up to six weeks depending on individual risk [14,20]. Building on Douillet et al.'s [22] general findings on lower limb immobilization, there is no clear benefit for prophylaxis beyond the immobilization period in low-risk patients.

## Conclusions

This review supports a risk-stratified, individualized approach to VTE prophylaxis in ATR patients. Utilization of standardized risk assessment tools such as TRiP(cast) score, alongside careful consideration of patient-specific factors, is essential to optimize outcomes while avoiding unnecessary treatment. Pharmacologic prophylaxis is indicated in high-risk patients, particularly post-surgical cases or those with multiple VTE risk factors. For low-risk patients, the benefit of LMWH or DOACs remains uncertain and inconsistently demonstrated across studies. In these patients, early mobilization, patient education, and mechanical prophylaxis may offer a safe alternative similar to the general population of lower-limb immobilized patients. Future high-quality prospective trials are needed to clarify prophylaxis efficacy, especially in the lower-risk ATR populations, and to evaluate the long-term safety of emerging agents like DOACs.

## Appendices

Appendix A 

**Table 6:** Search strategies

Database	Search Query	N
PubMed	((("achilles tendon"[MeSH Terms] OR ("achilles"[All Fields] AND "tendon"[All Fields]) OR "achilles tendon"[All Fields]) AND ("ruptur"[All Fields] OR "rupture"[MeSH Terms] OR "rupture"[All Fields] OR "ruptured"[All Fields] OR "ruptures"[All Fields] OR "rupturing"[All Fields])) OR (("achilles tendon"[MeSH Terms] OR ("achilles"[All Fields] AND "tendon"[All Fields]) OR "achilles tendon"[All Fields]) AND ("injurie"[All Fields] OR "injuried"[All Fields] OR "injuries"[MeSH Subheading] OR "injuries"[All Fields] OR "wounds and injuries"[MeSH Terms] OR ("wounds"[All Fields] AND "injuries"[All Fields]) OR "wounds and injuries"[All Fields] OR "injurious"[All Fields] OR "injury s"[All Fields] OR "injuryed"[All Fields] OR "injurys"[All Fields] OR "injury"[All Fields])) OR "ATR"[All Fields]) AND ((("venous thromboembolism"[MeSH Terms] OR ("venous"[All Fields] AND "thromboembolism"[All Fields]) OR "venous thromboembolism"[All Fields] OR "VTE"[All Fields]) AND ("VTE"[All Fields] AND ("prevention and control"[MeSH Subheading] OR ("prevention"[All Fields] AND "control"[All Fields]) OR "prevention and control"[All Fields] OR "prophylaxis"[All Fields] OR "prophylaxies"[All Fields] OR "prophylaxy"[All Fields]))) OR ("venous thrombosis"[MeSH Terms] OR ("venous"[All Fields] AND "thrombosis"[All Fields]) OR "venous thrombosis"[All Fields] OR ("deep"[All Fields] AND "venous"[All Fields] AND "thrombosis"[All Fields]) OR "deep venous thrombosis"[All Fields]) OR "DVT"[All Fields])	132
Scopus	TITLE-ABS-KEY ( ( ( achilles AND tendon AND rupture ) OR ( achilles AND tendon AND injury ) OR atr ) AND ( ( venous AND thromboembolism ) OR ( vte ) ( vte AND prophylaxis ) OR ( deep AND venous AND thrombosis ) OR ( dvt ) ) )	63
Web of Science	((Achilles tendon rupture) OR (Achilles tendon injury) OR ATR) AND ((Venous thromboembolism) OR (VTE) (VTE prophylaxis) OR (deep venous thrombosis) OR (DVT)) (All Fields)	109
Cochrane Library	(((Achilles tendon rupture) OR (Achilles tendon injury) OR ATR) AND ((Venous thromboembolism) OR (VTE) (VTE prophylaxis) OR (deep venous thrombosis) OR (DVT))):ti,ab,kw	39
Total= 343 Removed duplicates= 13 Title and abstract screening= 330 Full text screening= 15 Final included= 8		
